# Improving ADR reporting in Jordan: a qualitative exploration of pharmacists’ perspectives

**DOI:** 10.3389/fmed.2024.1513611

**Published:** 2025-01-06

**Authors:** Amal K. Suleiman

**Affiliations:** Department of Pharmacy Practice, College of Clinical Pharmacy, King Faisal University, Al-Ahsa, Saudi Arabia

**Keywords:** adverse drug reaction, community pharmacist, pharmacovigilance, medication safety, Jordan

## Abstract

**Background:**

Community pharmacists are most accessible to patients. Hence, they have a crucial role in ensuring drug safety by detecting and reporting adverse drug reactions (ADRs). However, there may be gaps in their knowledge of ADR reporting systems and barriers they face in reporting.

**Objective:**

This study aims to assess community pharmacists’ knowledge of ADR reporting systems in the Kingdom of Jordan, identify the barriers they face in reporting ADRs, and explore the broader factors that influence their involvement in pharmacovigilance activities.

**Methods:**

In-depth, semi-structured, face-to-face interviews were held with 20 community pharmacists from different regions of Jordan to evaluate their understanding of ADR reporting, the obstacles they encountered, and the elements that could motivate them to report ADRs. The interviews were transcribed and subjected to thematic analysis to find recurrent themes and insights. The thematic analysis highlighted opportunities for continuing education and an absence of formal training as the main barriers to ADR reporting.

**Results:**

Pharmacists reported dissatisfaction with time limits in their hectic work situations and the complexity of reporting procedures, especially the length and information demanded by ADR reporting forms. Another factor influencing low reporting rates was a perceived lack of acknowledgment and feedback. Participants proposed that encouraging ADR reporting with professional recognition or compensation and improving and digitizing the reporting process would promote increased participation.

**Conclusion:**

ADR reporting presents considerable difficulties for community pharmacists in Jordan, mostly because of administrative obstacles and an absence of official support and training. Enhancing pharmacovigilance efforts in Jordan could be achieved by providing incentives, simplifying the reporting procedure, and incorporating reporting into the current pharmacy management software.

## 1 Introduction

Adverse drug reactions (ADRs) pose a significant healthcare challenge due to the increasing complexity of therapeutic options, the aging global population, and the growing prevalence of comorbidities. Polypharmacy, especially in elderly patients, increases the risk of ADRs, making it harder to manage and monitor these patients effectively. The increasing complexity of healthcare elevates both the risk and impact of ADRs, emphasizing the need for dynamic risk management strategies (1). While randomized controlled trials (RCTs) are crucial for assessing drug efficacy and safety, their limited duration and homogeneous populations restrict their ability to monitor ADRs fully (2, 3). Consequently, continuous post-marketing surveillance is essential to optimize therapeutic benefits and confirm treatment effectiveness in a broader, more diverse patient population (4, 5).

Systematic reporting of adverse events during clinical trials is vital for building and updating each medication’s safety profile (6). Once a drug is on the market, healthcare systems increasingly rely on spontaneous ADR reporting to track safety, especially for uncommon or late-occurring ADRs (7). ADRs and adverse drug events (ADEs), as defined by the Institute of Medicine (IOM) pose a significant global health burden. ADEs refer to any harm caused by a drug-related medical intervention, including overdoses, allergic reactions, ADRs, and medication errors (8). ADRs in particular, notably contribute to morbidity and mortality (9, 10), highlighting the need for effective pharmacovigilance systems and continuous drug safety monitoring. Improved monitoring can reduce health risks by enabling early detection and prompt intervention. Research shows that hospitalization rates due to ADRs vary significantly between countries and regions. In Europe, rates can be as high as 12.8%, whereas in Australia, ADRs make up 2–12% of hospital admissions (11). Comparably, in the United Kingdom ADRs account for 6.5% of hospital admissions (12), and in Sweden, 12% of internal medicine department admissions are due to ADRs (13). Remarkably, ADRs were the sixth most common cause of death in industrialized nations, including the US, in 2002 (14). Furthermore, in a study of hospitalized patients between 2010 and 2019, significant reductions were noted in the annual rates of adverse events, including those due to ADRs, in conditions such as acute myocardial infarction, heart failure, pneumonia, and major surgeries (15).

Preventable ADRs occur at a rate of 37.9% in the Western region, highlighting their significant financial impact (16). A 2011 study by Paudel et al. (17) estimated that ADR-related hospitalizations in the US cost nearly US$38.9 billion. These figures underscore the urgent need for improved pharmacovigilance systems to reduce unnecessary patient suffering and the financial burden of inappropriate prescriptions (18). Most studies on post-marketing withdrawals are older, reflecting a gap in more recent research, and provide valuable insights into the patterns of drug withdrawals, it is important to note that the data is based on medicinal products withdrawn between 1950 and 2014, and the findings may be influenced by the period covered (19). ADRs, a leading cause of hospitalizations, morbidity, mortality, and delaying treatment also increase healthcare expenses (20). While regulatory bodies require marketing authorization holders to gather and share safety information (7), healthcare providers’ involvement is crucial, as patients are more likely to report ADRs to their physicians or pharmacists than to pharmaceutical companies. However, ADR reporting rates among healthcare practitioners remain low, primarily due to the lack of mandatory reporting in many countries, with up to 94% of practitioners in the European Union underreporting (21–23).

The Jordanian National Pharmacovigilance Center (JNPC) was established in 2001 by the Jordan Food and Drug Administration (JFDA), followed by the creation of the Jordan Pharmacovigilance Centre (JPC) in 2002 to gather and assess ADR reports. In 2006, ADR guidelines were introduced based on International Conference on Harmonization (ICH) standards. The JPC system is accessible to healthcare professionals, enabling timely interventions to protect patient safety. Despite efforts to decentralize pharmacovigilance activities with regional centers established between 2011 and 2015, a study by Onakpoya et al. (19) found only 428 ADR reports submitted to the JFDA, indicating underreporting. This gap was due to limited understanding and engagement with pharmacovigilance principles among healthcare professionals (24). The study emphasized the need for better educational initiatives to encourage ADR reporting.

A 2023 study by Mhaidat et al. (25) analyzed ADR reports submitted to the pharmacovigilance database of the JFDA from 2015 to 2021 revealing a total of 2,744 reports. Despite broad data collection, community pharmacists contributed only a small percentage of the total reports, with 28.4% of these classified as serious. The most common drug types associated with ADRs were antineoplastic and immunomodulating therapies (24.0%), systemic antibiotics (14.2%), and gastrointestinal and metabolic medications (12.1%). Interestingly, with 22.8% of reports, COVID-19 vaccines were the most often reported item. Fatigue (6.3%), injection site pain (6.1%), and headache (6.0%) were the most frequent particular ADRs. 4.7% of the ADRs for which outcome data was available had a fatal outcome. The consistently low rates of ADR reporting pose a serious healthcare challenge, delaying regulatory actions to restrict unsafe medications. Under-reporting, however, is still a major problem in Jordan and is linked to several issues, including inadequate training, a lack of awareness of the reporting system, and a reliance on other medical professionals to report (4, 5, 10).

In Jordan, community pharmacies follow a fee-for-service model, where pharmacists are paid for medications. However, there is limited financial support for clinical services, including ADR reporting, and reimbursement policies do not cover ADR reporting or patient counseling. These limitations can impact pharmacists’ motivation and participation in ADR reporting systems (26). Pharmacists’ reporting of suspected ADRs is a critical component of Jordan’s pharmacovigilance system, led by the JFDA and the Rational Drug Use & Pharmacovigilance Department. These reports even those with a limited number of incidents are essential for spotting any drug safety concerns and improving the country’s ADR database. However, there is a lack of published studies addressing the understanding and challenges of ADR reporting among community pharmacists in Jordan (27). Prior studies indicate that many pharmacists are unaware of the ADR reporting system (25). Effective pharmacovigilance is key to identifying the risks of new medications, supporting evidence-based prescribing, preventing adverse reactions, and optimizing patient therapy at reduced costs. This study aims to explore community pharmacists’ knowledge and barriers to ADR reporting in Jordan, emphasizing their crucial role in ADR detection, improving post-marketing surveillance, and enhancing pharmacovigilance awareness among healthcare providers in community pharmacies.

## 2 Methods

### 2.1 Study design

This qualitative study aimed to explore the perspectives of Jordanian community pharmacists on their knowledge and experiences with reporting Adverse Drug Reactions (ADRs). The primary goal was to gain in-depth insights into pharmacists’ awareness of pharmacovigilance, barriers to ADR reporting, and the potential facilitators that could encourage greater participation in ADR reporting systems. The research employed an exploratory qualitative design, which was conducted between August 2023 and February 2024.

### 2.2 Sampling technique

A purposive sampling technique was employed to ensure a broad representation of experiences and viewpoints. Pharmacists with 7–10 years of experience, primarily working in chain pharmacies across northern, central, and southern regions of Jordan, were selected. This approach was intended to capture a wide range of demographic diversity and professional experiences relevant to the study. The sample size was determined by data saturation, meaning no new insights were gained after a certain number of interviews.

### 2.3 Data collection

Data were collected through semi-structured, in-depth interviews conducted in-person, allowing flexibility in capturing participants’ perspectives on ADR reporting. Each interview lasted between 30 and 45 min, conducted in locations convenient to the participants to accommodate their schedules and preferences. With the participants’ consent, all interviews were audio-recorded to ensure accurate data capture.

The interview guide, developed after reviewing the literature on pharmacovigilance and ADR reporting, included open-ended questions regarding pharmacists’ knowledge of ADRs, barriers to reporting, and recommendations for improvement. After the interviews, participants were allowed to review the recordings and transcripts for accuracy.

### 2.4 Data analysis

Data were analyzed using inductive thematic analysis, a widely recognized method in qualitative research for identifying patterns and themes within textual data (28). The analysis process began with a verbatim transcription of the interview. The interviews were transcribed verbatim by a member of the research team. This was done manually. To ensure a thorough understanding of the data, the research team listened to the audio recordings multiple times and reviewed the written transcripts repeatedly. This immersion in the data helped the researchers in understanding the data. The research team, consisting of experts in pharmacy practice, pharmacovigilance, and qualitative research, systematically coded the data, extracting relevant details from each interview and applying specific codes to capture key elements of participants’ responses. These codes were refined through discussions within the research team to ensure consistency and relevance. The team was selected based on their expertise in community pharmacy and ADR reporting systems, ensuring the analysis was informed by their professional backgrounds. However, their familiarity with the subject matter may have influenced the interpretation of the findings, so cross-checking was performed at every stage to ensure the credibility and reliability of the results. After repeated readings, the team organized the codes into broader themes, minimizing redundant codes and refining the structure to ensure each theme accurately reflected the data. Throughout the analysis, cross-checking was performed at every stage to ensure the credibility and reliability of the findings, enhancing the consistency and rigor of the thematic analysis (29).

### 2.5 Ethical considerations

Ethical approval for the study was secured from the Research Health and Medical Committee under reference R0002412, and all participants provided informed consent before taking part in the study. Participants were assured that their involvement was voluntary and that all information would be treated confidentially.

## 3 Results

Table 1 shows the demographics of participants. A total of 20 pharmacists participated in the study whose mean age was 28. Fifty percent of the participants were female, and 50% of them put in more than 40 h a week at work. Seventy five percent of the group had a bachelor’s degree, and 45% had 5–10 years of experience. There is a notable discrepancy between ADR observation and reporting while 90% of respondents had seen less than five ADR incidents, only 5% had reported any ADRs in the previous year.

**Table 1:** Demographics of participants.

Variable	*n* = 20 (%)
Age	Mean = 28 years ±2.7 range (23–36 years)
**Gender**	
Male	8 (40.0)
Female	12 (60.0)
**Hours worked per week**	
<24 h	1 (5.0)
24–48 h	9 (45.0)
>48 h	10 (50.0)
**Highest level of qualification**	
Bachelors	15 (75.0)
Post-graduate	5 (25.0)
**How long have you been working as a pharmacist?**	
0–5 years	7 (35.0)
5–10 years	9 (45.0)
More than 10 years	4 (20.0)
**How many adverse medication reaction (ADR) cases have you observed in your present practice?**	
Less than 5	18 (90.0)
6–12	1 (5.0)
More than 12	1 (5.0)
**In the past year, have you ever reported any ADRs that you have observed in your patients?**	
Yes	1 (5.0)
No	19 (95.0)

The qualitative investigation highlights important challenges and obstacles that Jordanian community pharmacists encounter while reporting ADRs. These barriers can be categorized into five primary themes: Lack of Awareness and Training, Complexity of Reporting Procedures, Perceived Impact and Motivation, Professional Environment, and Suggestions for Improvement. These themes are discussed below, with supporting categories and participant quotations.

### 3.1 Theme 1: lack of awareness and training

Pharmacists described gaps in both their initial education and ongoing professional development concerning ADR reporting, impacting their ability to effectively participate in the process.

#### 3.1.1 Insufficient formal training

Participants reported not receiving formal instruction on ADR reporting during their academic training. They also expressed that additional emphasis on ADR reporting in the curriculum would be beneficial.

*“I have not received any formal training on how to report ADRs. Everything I know has been picked up informally, through trial and error.” (Pharmacist: 2) “ADR reporting was mentioned during pharmacy school but wasn’t covered in sufficient depth to navigate the reporting process properly.” (Pharmacist: 4).*

#### 3.1.2 Lack of continuing education

Participants also emphasized the lack of possibilities for ADR-focused continuing education. This suggests that there is a need for continued professional development so that pharmacists are aware of changing medication safety regulations and reporting guidelines.

*“There aren’t any training or revision sessions on ADR reporting accessible for us. We require constant updates.” (Pharmacist: 1).*

*“Continuous pharmacovigilance education should be provided to pharmacists. It would assist us in remaining up to date on new medications and reporting guidelines.” (Pharmacist: 7).*

### 3.2 Theme 2: complexity of reporting procedures

Pharmacists noted that the ADR reporting process was complicated and time-consuming, with multiple barriers hindering their willingness to report ADRs.

#### 3.2.1 Lengthy and complex forms

Participants reported that the forms required for ADR reporting were often too detailed and required information that was difficult to obtain in a busy pharmacy environment.

*“The reporting forms ask for so much knowledge that I cannot usually provide, like details from the patient’s medical history.” (Pharmacist: 5).*

*“With my hectic schedule, I do not have time to fill out this lengthy paperwork. They ask for far too much information.” (Pharmacist: 6).*

#### 3.2.2 Time constraints

Due to the high demands of patient care, many pharmacists explained that finding the time to fill out ADR reports was challenging.

*“I am always occupied with patients, and it takes too long to complete an ADR report. People are constantly in line.” (Pharmacist: 17).*

*“In our pharmacy, we are overloaded with duties, and ADR reporting is a low concern compared to urgent patient care.” (Pharmacist: 5).*

#### 3.2.3 Lack of system integration

Pharmacists suggested that the lack of integration between ADR reporting and their current pharmacy management software created an additional layer of complexity and reduced their likelihood of reporting ADRs.

*“If ADR reporting had been built into the software that we employ for dispensing, I would be much more likely to report.” (Pharmacist: 3).*

*“A direct link among pharmacy management programs and the ADR report system would make the procedure simpler and faster.” (Pharmacist:8).*

### 3.3 Theme 3: perceived impact and motivation

Pharmacists expressed that the lack of feedback and recognition for their ADR reports diminished their motivation to participate in the process.

#### 3.3.1 Lack of feedback and visibility

Participants mentioned that after submitting ADR reports, they rarely received any feedback, leaving them unsure about the outcomes or effectiveness of their submissions.

*“I have previously reported a few ADRs, but I never received a response. I’m not sure if my reports had any impact.” (Pharmacist: 17).*

*“Without knowing the outcomes of our reports, it feels like a futile exercise.” (Pharmacist:9).*

#### 3.3.2 Limited recognition

Pharmacists felt that there were no incentives or recognition for reporting ADRs, which contributed to their reluctance to engage in the process.

*“There’s no incentive to report ADRs. It’s just extra work with no benefit for us.” (Pharmacist:18).*

*“If there were rewards like professional points or certificates, I would be more motivated to participate in ADR reporting.” (Pharmacist: 5).*

### 3.4 Theme 4: professional environment

The professional environment, including peer support and encouragement from supervisors, was seen as an important factor in influencing ADR reporting practices.

#### 3.4.1 Lack of peer support

Many participants indicated that there were limited opportunities to discuss ADR reporting with colleagues, which could help increase their confidence and understanding of the process.

*“I would like there was a way to talk about ADRs with my colleagues, perhaps an online group or regular meetings.” (Pharmacist: 14).*

*“Having peer support would help us feel more confident in reporting ADRs.” (Pharmacist: 15).*

#### 3.4.2 Lack of supervisory encouragement

Some pharmacists mentioned that supervisors did not emphasize the importance of ADR reporting, instead focusing on other priorities such as sales targets.

*“ADR reporting is never brought up by my supervisor; we are only concerned with achieving our sales goals.” (Pharmacist: 20).*

*“Patient safety is important, but it becomes an afterthought when management does not emphasize it.” (Pharmacist: 16).*

### 3.5 Theme 5: suggestions for improvement

Pharmacists provided several suggestions for improving ADR reporting, including simplifying the reporting process, integrating it with existing systems, and offering incentives for participation.

#### 3.5.1 Simplification of forms

Participants suggested that simplifying the forms and digital reporting system could make ADR reporting less burdensome and more efficient.

*“A simple, digital form that I can fill out quickly on my phone or computer would make a big difference.” (Pharmacist: 11).*

*“Digitalizing the reporting process would reduce the burden and make it easier for us to report ADRs.” (Pharmacist: 1).*

#### 3.5.2 Integration with software

Many pharmacists recommended that ADR reporting should be integrated into existing pharmacy management systems to streamline the process and save time.

*“If the system we use for dispensing could also be used for ADR reporting, it would be so much easier.” (Pharmacist: 7).*

*“Electronically integrating patient information into the ADR reporting form could save us a lot of time.” (Pharmacist: 13).*

#### 3.5.3 Incentives and recognition

Pharmacists felt that offering rewards or recognition for ADR reporting would boost participation. *“It takes a lot to get recognition. We would feel that our efforts are appreciated even if we only received a certificate or other recognition.” (Pharmacist: 20).*

*“Benefits, even small ones, would significantly boost our willingness to participate in ADR reporting.” (Pharmacist: 17).*

## 4 Discussion

This study offers insightful information about the attitudes, practices, and knowledge of Jordanian community pharmacists about reporting ADRs. According to the results, the majority of participants did not have the necessary knowledge or formal training to report ADRs. This indicates that there are still significant weaknesses in the nation’s pharmacovigilance education system. The JNPC and ADR reporting are not well known, which indicates that there is still more effort to be done to incorporate ADR reporting into standard pharmacy practice. This lack of awareness mirrors findings from previous studies (30), where over half of the health professionals were similarly uninformed about their respective national pharmacovigilance programs (31). Comparable studies in Saudi Arabia have also highlighted a deficiency in knowledge among health professionals, despite generally positive attitudes toward ADR reporting. This includes limited awareness of the various types of ADRs, such as those related to antibiotics, herbal medicines, and vaccines, which require specific attention and reporting protocols (32). Similarly, in Turkey, ignorance of the national pharmacovigilance system has been cited as a primary cause for the under-reporting of ADRs (33). These observations unequivocally suggest that there is a pervasive global trend of inadequate awareness regarding spontaneous reporting systems among health professionals, necessitating strategic interventions to bridge this knowledge gap.

The study examining pharmacists’ attitudes toward ADR reporting in Jordan highlights several factors influencing their reporting practices, including legal obligation, regular guidelines, feedback from authorities, simple reporting methods, and patient requests. Addressing these issues collectively could enhance ADR reporting efficacy and improve patient safety outcomes. As a result, these pharmacy owners may not emphasize the idea of services or reporting ADRs, which is probably the situation in many underdeveloped nations (4, 34).

In the examination of barriers to ADR reporting among pharmacists, the primary obstacle identified was a lack of knowledge about how to report, which significantly hinders their participation in pharmacovigilance. This is compounded by systemic issues such as the absence of reporting forms and their absence of integration with the JNPC system, which was the second most significant barrier. Furthermore, the pharmacists reported that the process of reporting ADRs is problematic, aligning with commonly cited barriers such as the perceived complexity of the reporting procedures. Additionally, the time required to complete reports was also a notable deterrent, reflecting the broader challenge of time constraints faced by healthcare professionals. Consistent with findings from prior research, which identified logistical challenges as the main deterrents to community reporting of ADR pharmacies within the same region, this study also highlights similar issues (35). However, it additionally reveals that community pharmacists exhibit both enthusiasm and confidence in their ability to report and categorize ADRs, aiming to improve patient welfare.

In Jordan, the community pharmacy sector is predominantly business-oriented, which may result in the relegation of ADR reporting and service provision to a lower priority by pharmacy entrepreneurs. This issue of priority aligns with international observations; for instance, community pharmacists in Hong Kong, Holland, and the UK have shown an absence of awareness about the ADR reporting programs in their respective countries (36–38). Furthermore, the effectiveness of regulatory authorities in enforcing ADR reporting regulations in community pharmacy practice remains a concern, highlighting a potential gap in regulatory oversight. The study participants all agreed that reporting ADRs could improve drug safety, but they did not see a difference in the quality of life for patients who regularly visited community pharmacies. To maintain a strong ADR reporting system, routine inspections by Jordan’s health authorities are considered vital to guarantee medication safety and detect possible drug-related dangers within the Jordanian population. Furthermore, requiring pharmacists to complete ADR reporting training before receiving their licenses might significantly raise the knowledge of Jordan’s ADR reporting procedures among pharmacists working both domestically and abroad. The entire healthcare system depends on community pharmacies having a methodical approach to ADR reporting. To promote a culture of spontaneous ADR reporting in Jordan, pharmacists should get frequent training and ongoing education (39, 40). The use of incentives, including monetary prizes, may improve the effectiveness of ADR reporting even more (37, 39). Additionally, community pharmacies that have internet access may be able to report ADRs online to drug regulatory bodies, which would simplify the procedure and possibly improve compliance and efficacy.

Pharmacists have not, as anticipated, made a substantial contribution to ADR reporting. This observation is consistent with research by El-Dahiyat et al. (27), who pointed out that pharmacists have not done much in this field. This result is especially unexpected since, as prior studies have shown, pharmacists are considered to be specialists in drug information and are essential in maximizing medicine therapy (41, 42). Furthermore, research has shown that pharmacist-led healthcare interventions can improve the caliber of ADR reporting while dramatically lowering pharmaceutical and prescribing errors (43, 44). Therefore, it improves Jordanian pharmacists’ ability to identify, track, and report ADRs.

## 5 Limitations

This study provides important insights into the views of community pharmacists in Jordan on ADR reporting, but several limitations must be considered. First, the sample size was relatively small and may not fully represent the broader population of pharmacists across Jordan. As a result, the findings reflect the experiences of those who participated and may not generalize to all pharmacists in the country. Second, the study may be influenced by personal biases. Participants may have responded in a way they perceived regarding their knowledge and practices related to ADR reporting. Additionally, the study utilized qualitative methods (such as interviews). The data captured is specific to the themes identified within the sample, and other factors influencing ADR reporting may not have been fully explored. Lastly, the study did not investigate structural or organizational factors within the healthcare system that might also affect ADR reporting. Future studies could consider exploring these broader contextual factors to provide a more comprehensive understanding of the challenges and opportunities in improving pharmacovigilance in Jordan.

## 6 Conclusion

This study provides insights into the perceptions of Jordanian community pharmacists regarding ADR reporting, highlighting areas where improvements in training and awareness could be made. While pharmacists generally have a positive attitude toward pharmacovigilance, many are not sufficiently trained or knowledgeable about the ADR reporting system. The results suggest that ADR reporting is underutilized, with barriers such as lack of motivation, complex procedures, and time constraints identified as key factors. These findings point to the potential benefits of simplifying the reporting process, enhancing pharmacist education, and incorporating ADR reporting into pharmacy management systems. Additionally, providing incentives and offering continuous training may help overcome these challenges. Addressing these barriers is crucial for improving pharmacovigilance and ensuring better patient safety outcomes in Jordan.

## Data Availability

The original contributions presented in the study are included in the article/supplementary material, further inquiries can be directed to the corresponding author.
